# Metastatic Tumour to the Mandible - A Diagnostic and Management Dilemma

**DOI:** 10.7759/cureus.5093

**Published:** 2019-07-07

**Authors:** Rinku George, Mahathi Neralla, Jyotsna Rajan, Ahmed Elham Haque, Santhosh P Kumar

**Affiliations:** 1 Oncology, Oral Cancer Institute, Saveetha Dental College, Chennai, IND; 2 Oral and Maxillofacial Surgery, Saveetha Dental College and Hospital, Saveetha University, Chennai, IND

**Keywords:** primary tumour, metastasis, lung, metastatic carcinoma, intraosseous carcinoma, mandible

## Abstract

Metastatic tumours of the jaw are overlooked due to their relatively rare incidence. However, they are often the first indicators of an unknown primary malignant lesion. In this case report, we present a 68-year-old male patient with a suspected intraosseous malignancy of the mandible who was treated by a right segmental mandibulectomy. The final histopathology report was indicative of a secondary metastatic tumour. Positron emission tomography scan revealed a suspicious lesion in the right lung, which was identified as the primary tumour by biopsy using the Tru-Cut® biopsy device (MeritMedical, Jordan UT). The metastatic lesion to the oral soft tissues was easily recognized, in contrast to the jawbone metastasis. Differentiating between primary intraosseous and metastatic mandibular tumours relies on the histopathologist and the surgeon working in tandem to arrive at an early conclusive diagnosis. Knowledge of metastatic tumours to the facial bones is indispensable to a surgeon as it can often be the first indication of an unknown primary malignancy. Identification of early signs, appropriate and timely investigative procedures, coordination between pathologist and surgeon, and choosing the correct treatment modality can help prolong and improve the quality of life of the patient.

## Introduction

Mandibular swelling is often the first symptom for pathologies associated with the mandible. Diagnosis of swellings without a clear-cut etiology can be quite challenging. In such cases, eliciting an accurate and relevant history from the patient will help in diagnosing the lesion. The orthopantomogram is a useful investigation aid for diagnosis, as most pathology is represented in a very characteristic manner. Swellings with infectious etiology are easier to rule out than non-infectious swellings. Non-infectious swellings may present with or without pain, have a moderate to rapid growth pattern, neurological disturbance, superinfection, and bleeding. Pain has varied manifestations; the patient may present with a painful benign tumour or a painless malignant growth. In these cases, odontogenic tumours, such as ameloblastoma, odontogenic myxoma, keratocystic odontogenic tumour, central giant cell granuloma, aneurysmal bone cyst, central arteriovenous malformations (AVM), intraosseous squamous cell carcinoma, and metastatic carcinoma, should be considered for differential diagnosis [1].

A well-defined radiolucent lesion is first aspirated to rule out AVM, followed by an intraosseous biopsy to establish a diagnosis. If the histopathological diagnosis is inconclusive, the aspirated content will be a valuable adjunct for diagnosis. In this case report, we discuss the challenges in establishing a diagnosis, treatment planning, and surgical decision-making for an ambiguous mandibular swelling.

## Case presentation

A 68-year-old male presented to the oral oncology outpatient department with a swelling over the right side of the mandible of one month's duration. A comprehensive history was taken, and the patient revealed a history of extraction of multiple teeth on the same side, after which the swelling started insidiously. It was soon accompanied by intermittent episodes of pain, which was of a pricking nature and radiated upwards along the jaw and to the ear on the same side. The patient was prescribed analgesics and anti-inflammatory medications, which provided temporary pain relief but there was no decrease in the swelling. The patient also complained of excessive salivation for two weeks. The patient had a habit of smoking 10 - 15 cigarettes a day for more than 25 years with occasional alcohol consumption. There was a negative history of chewing tobacco in any form.

Clinical examination revealed a bony hard swelling of the right side of the mandible, approximately 5 x 4 cm in maximum dimension, extending from the angle of the mandible to 2 cm short of the midline. The swelling was tender on palpation, with defined edges, and was fixed over the bone. No crackling or crepitus was elicited and the overlying skin was unremarkable. However, paraesthesia of the skin over the ipsilateral chin and lower lip was elicited.

**Figure 1:**
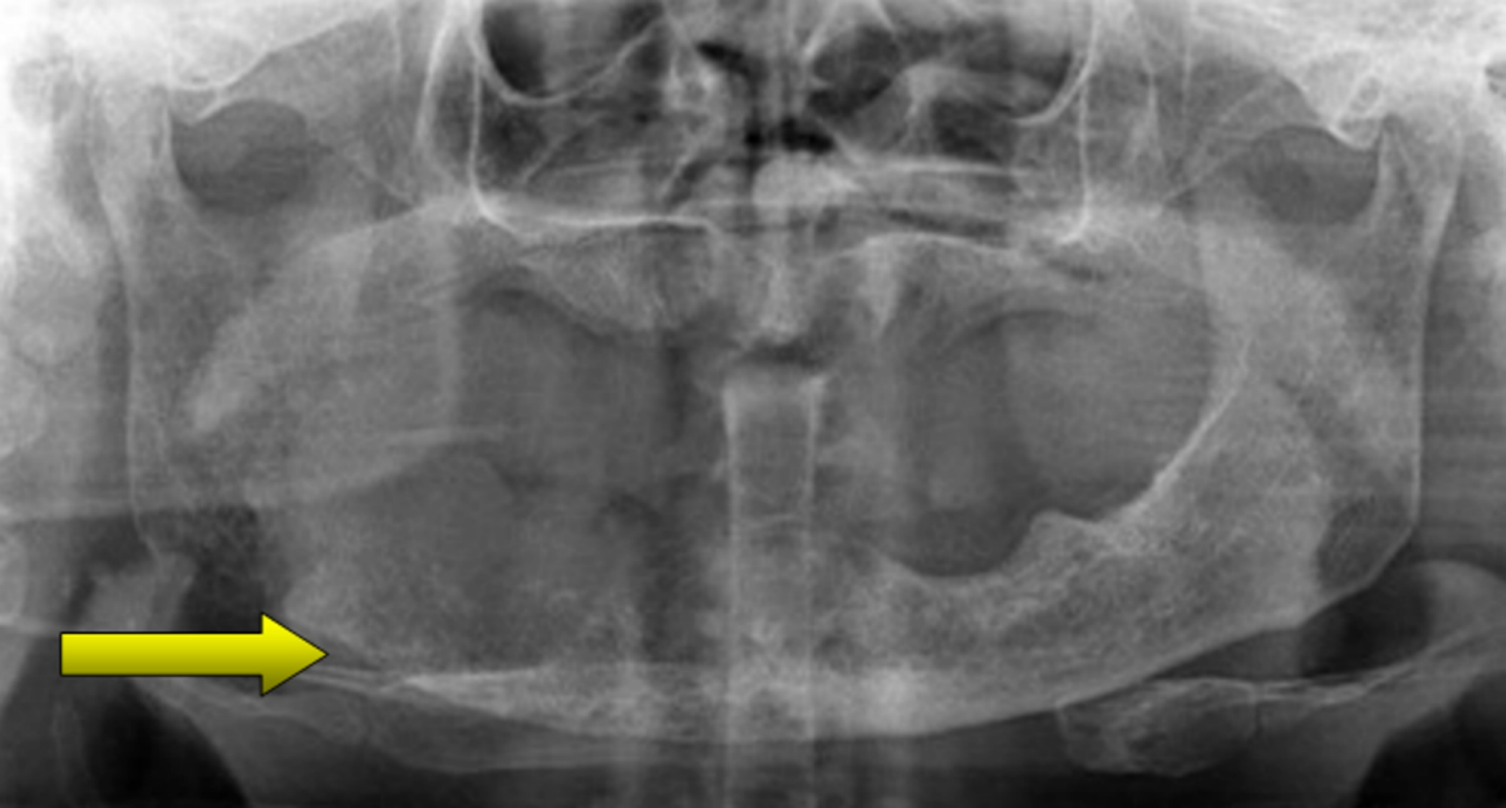
Preoperative orthopantomogram showing radiolucent areas in the right body and ramus of the mandible indicating erosions

On intraoral examination, no abnormality of the oral mucosa was detected and the patient was edentulous. Tenderness was elicited on palpating the alveolar mucosa on the right side, up to the retromolar region. The neck was palpated; however, no significant cervical nodes were found. An orthopantomogram was taken which showed a large radiolucency with irregular borders extending from the lower right canine region to the right angle of the mandible (Figure 1).

Clinical findings and previous biopsy reports were correlated and the diagnosis was inconclusive. The lesion was provisionally diagnosed as an intraosseous malignancy of the mandible. The oral pathologist suggested an excisional biopsy for confirmation of the lesion and the patient was planned for surgery. 

**Figure 2:**
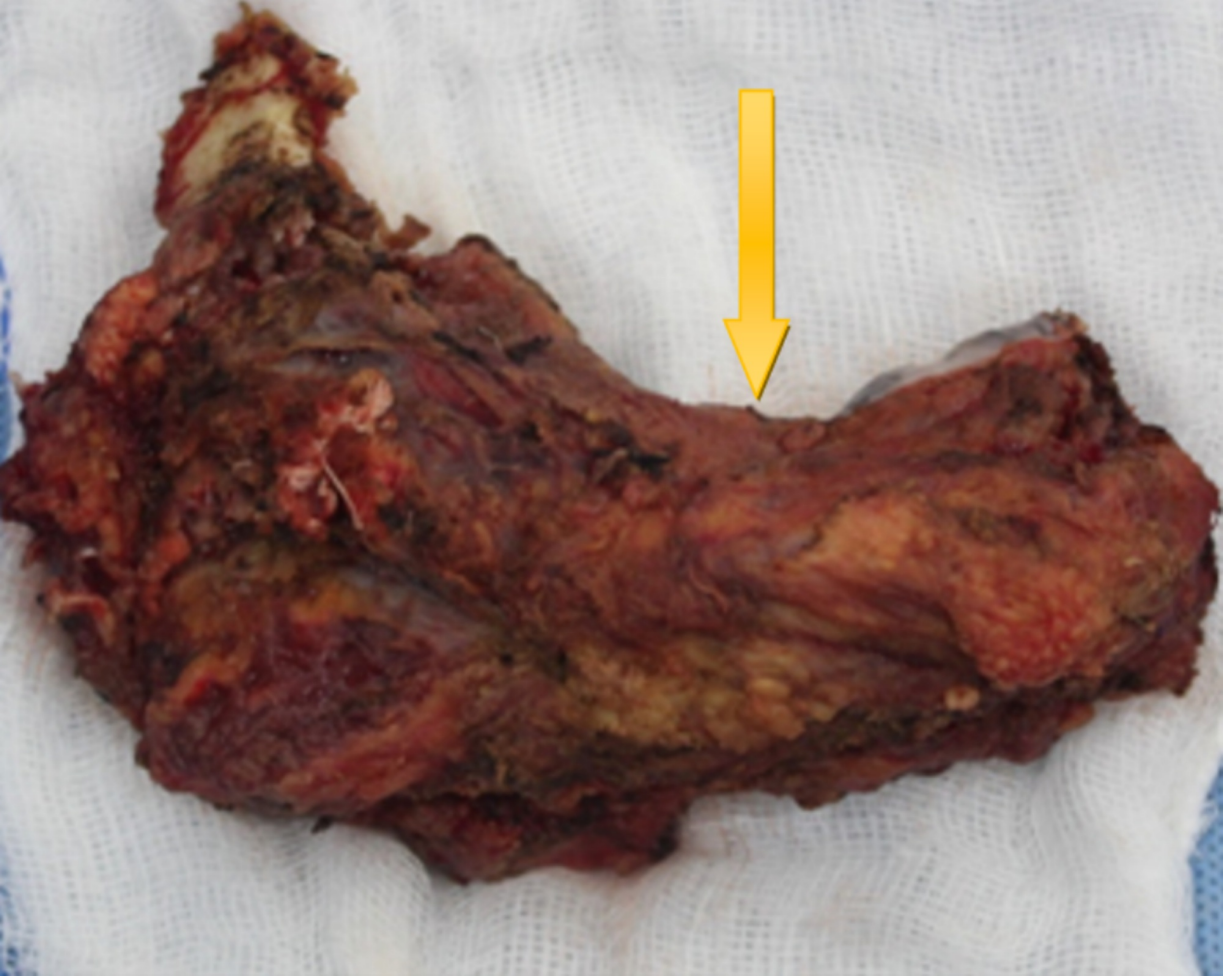
Resected mandibular specimen from the right side

The proposed treatment plan was a segmental mandibulectomy of the affected area and intraoperative frozen section, followed by reconstruction using a reconstruction plate. Segmental resection of the mandible from 42 regions up to the right sub-condylar region was performed under general anaesthesia, and the intraoperative frozen section finding was still inconclusive (Figure 2).

**Figure 3:**
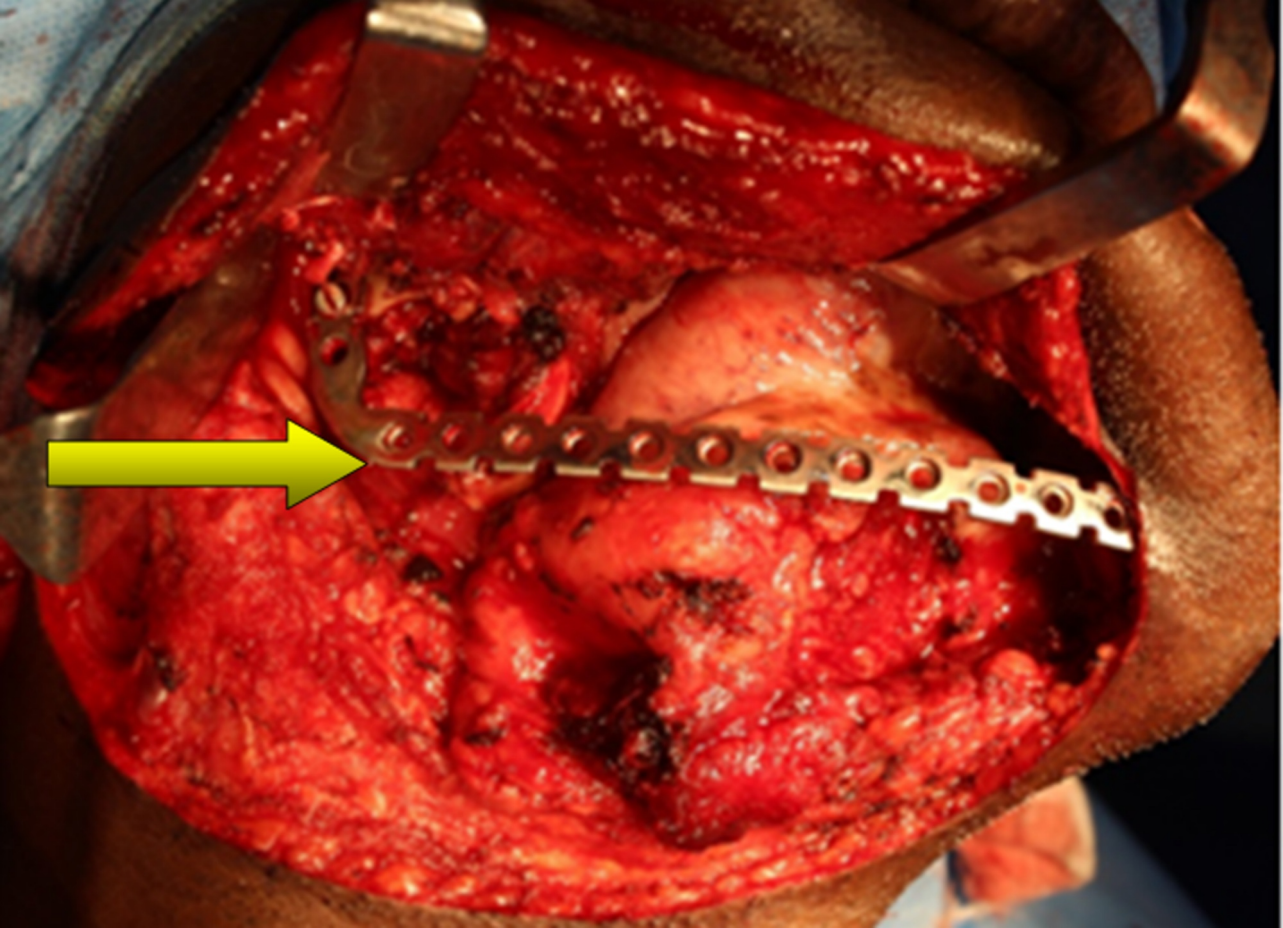
Reconstruction of the surgical defect with a stainless steel reconstruction plate

The excised specimen was sent for histopathological examination. Reconstruction was carried out with a contoured stainless-steel reconstruction plate and a watertight primary closure was achieved (Figure 3).

The postoperative period was initially uneventful. However, on the fifth postoperative day, the patient had a purulent discharge from the submental region, with gaping of the surgical wound at the retromolar region intraorally. Culture and sensitivity testing from swabs taken were positive for Pseudomonas growth, and intravenous antibiotics were prescribed accordingly. With antibiotic therapy, the patient recovered from the infection and there was satisfactory wound healing.

**Figure 4:**
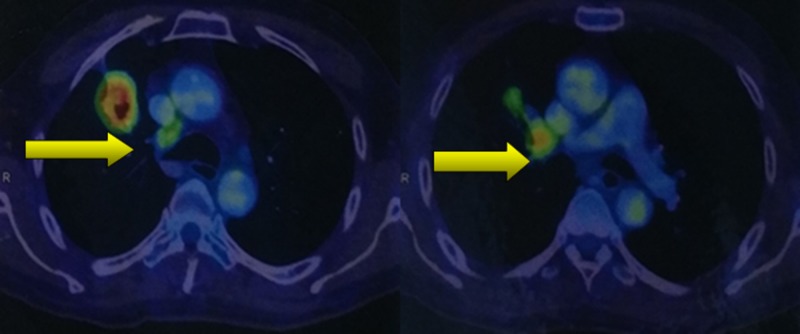
Positron emission tomography/computed tomography (PET/CT) scan showing cavitary lesions in the right lung, indicating the site of the primary tumour

The final histopathology report was suggestive of a high-grade malignancy of epithelial origin (metastatic adenocarcinoma/renal cell carcinoma). Positron emission tomography (PET) scan revealed well-defined, thick-walled cavitary lesions in the superior segment of the right upper lobe and the anterior segment of the right lower lobe of the lung (Figure 4).

The patient was referred to an interventional radiologist and a Tru-Cut® (MeritMedical, Jordan, UT) biopsy was performed, which confirmed the malignant growth in the right lobe of the lung. The patient is currently undergoing palliative chemoradiotherapy for the primary tumour of the lung (oral Gefitinib, 250 mg, and 10 fractions of radiotherapy).

## Discussion

Metastasis refers to the development of secondary cancerous implants, discontinuous with the primary tumour, and possibly in remote tissue [2]. Metastatic tumours to the orofacial region are relatively uncommon, with only 1% - 3% of head and neck cancers originating from distant sites [3]. Of these, the jawbones are affected twice as often as the oral soft tissues [4]. The mandible is more susceptible to metastatic deposits, with incidences varying from 69% to 82% [4-6]. Very often, metastases to the oral region are the first sign of metastatic spread (25%) [4], and more significantly, it can be the first indication of an undiscovered distant primary tumour (23% - 62%) [4, 6].

The metastatic process is a complex biological cascade, and the cellular basis of site-specific tumour metastasis was first described by Paget [7] in 1889 with his “seed and soil” hypothesis and later by Zetter [8] in 1990. This theory inferred that certain tumours had the propensity to spread to certain tissues which had the ability to support the growth of the tumour cells. Later, Ewing [9] proposed the “mechanical theory” of metastasis, attributing the spread of tumours entirely via blood flow carrying the tumour cells away from the primary site. The secondary sites were passive receptacles to the tumour emboli. However, tumor metastasis is a combination of the two theories, where neoplastic cells travel through the vascular or lymphatic system, implant into the new tissue, and thrive while fighting the body’s natural defense systems.

Hart [10] stated that site-specific metastasis aided this process, and growth factors released from certain tissues could aid the growth of certain tumour cells while causing an inhibitory effect on another. The spread of tumour emboli can occur through the lymphatics, blood vessel permeation, transcoelomic permeation, local infiltration, or a combination of them [2]. With respect to the head and neck, the Batson venous plexus, a valveless vertebral venous plexus that bypassed the filtration of the lungs, became an established route of metastasis from the gastrointestinal, genitourinary, and respiratory systems [11].

In a retrospective analysis of metastatic tumours to the oral cavity by Hirshberg et al. [4], the lung was the most common primary site affecting the jawbones in men (22%) and the breast in women (41%). This has been corroborated over the years by numerous studies [3, 5-6, 12-13]. Similarly, in our case, the metastasis to the mandible was from the lung tumour. Mandibular bone, although not rich in red marrow, is prone to metastasis, with the molar region most commonly involved, followed by the premolar area and ramus-angle region [14]. Hashimoto et al. [15] suggested that the early formation and presence of haematopoietically active sites could act as foci for the secondary deposits. However, the pathogenesis of metastasis to the jawbones is still not clear.

A metastatic lesion to the oral soft tissues is easily recognized, in contrast to jawbone metastases. By the time any involvement of the bone becomes evident to the naked eye, the disease has probably disseminated and the survival of the patient is diminished. In the jawbones, most patients complain of swelling, pain, and paraesthesia which develop in a relatively short time [16]. Pain is the first presenting symptom in most cases. Numb chin syndrome or mental neuropathy is a unilateral sensory disturbance of the lower lip, chin, and/or the gingival mucosa which should always raise suspicion for metastatic disease to the mandible. In such cases, an osteolytic lesion with poorly-defined margins is the commonest radiographic presentation [3]. Our patient had intermittent pain and nerve disturbances in the site of the mandibular lesion.

The initial workup of patients presenting with a presumed cancer of unknown primary should not be exhaustive and should instead be focused on the evaluation of likely primary sites. A thorough history and physical examination, complete blood count, urine analysis, biochemistry, chest radiography, and computed tomography of the lung, abdomen, and pelvis should be performed [6]. PET scanning with fluorodeoxyglucose (FDG) is rapidly gaining favour in the evaluation of unknown primary cancers, particularly in instances where other imaging modalities have failed to identify a source [4].

The histopathological diagnosis is key in evaluating patients with cancer of an unknown primary. Communication between the surgeon and pathologist is essential. Ideally, the pathologist can identify the origin; however, it might be impossible in some cases owing to the rapid mitotic growth of the cells, making it indistinguishable [6]. In our case, it was initially difficult to diagnose the lesion from the incisional biopsy. However, the histopathological examination of the excised specimen revealed metastatic adenocarcinoma, with the commonest site of origin in males being the lungs. Serum markers, although they lack adequate specificity, can be used alongside the pathological and clinical information and may be helpful in diagnosing the origin of the lesion [17].

Bronchogenic carcinoma is one of the most lethal malignancies worldwide. Approximately 9% - 30% of patients with lung cancer develop bone metastases, leading to significant morbidity and mortality. Lung carcinomas are characterized by their insidious onset, difficulty in detection, early metastatic spread, and poor prognosis at the time of presentation [18]. In our case, the metastatic growth in the mandible was from primary lung cancer and it was detected by PET scan. Atypical facial pain, not attributable to malignancy, was a unique feature described by Abraham et al., with the onset of pain preceding the other symptoms of malignancy by one to 48 months [19].

The prognosis for survival upon the discovery of maxillofacial metastasis is poor [3-4, 20] with as little as four months [6]. With the extensive nature of the metastatic disease, these patients classically have been deemed as nonsurgical candidates. Our patient is currently undergoing palliative chemoradiotherapy. In some cases where the oral metastasis is the only metastatic site, adequate surgical treatment with or without radiotherapy can improve morbidity, although with only palliative benefits [3, 16]. Surgical excision can also play a beneficial role in improving the quality of life, which is an important outcome assessment in head and neck cancer patients.

Oral metastatic lesions can often cause progressive discomfort, bleeding, superinfection, dysphagia, interference with mastication, and disfigurement. Thus, it directly affects social, functional, and psychological aspects of life and is often the only concern for the patient [16]. With advances in the molecular biology of metastasis, new insights and substantial efforts are being made to uncover new therapeutic modalities targeting cancer cells, even in widespread diseases. The reality, however, is that palliative care with radiotherapy or chemotherapy, and in very select cases, surgery, is still the present-day treatment strategy.

## Conclusions

The diagnosis of metastatic lesions in the oral cavity is a challenge due to its rarity. While one cannot be overzealous, a multidisciplinary approach to swiftly identify the metastasis can be of significance to its treatment, as well as discovering the primary site. Fine needle aspiration cytology/biopsy is the gold standard for characterization of the tumour, and role of the pathologist in hastening the discovery of the primary is invaluable. Sequential investigations, such as plain radiographs, complete blood picture, biochemical investigations, computed tomography, magnetic resonance imaging, and PET scan, when correlated with clinical findings, will confirm the diagnosis. Palliative radiation therapy and/or chemotherapy is the preferred modality of treatment. Surgical intervention to reduce the suffering of the patient and to improve the quality of life should be tailored to each case with careful patient selection. 
